# Selective Separation of Chromium Species from Soils by Single-Step Extraction Methods: a Critical Appraisal

**DOI:** 10.1007/s11270-017-3459-5

**Published:** 2017-07-13

**Authors:** Barbara Leśniewska, Marta Gontarska, Beata Godlewska-Żyłkiewicz

**Affiliations:** Institute of Chemistry, University of Bialystok, K. Ciołkowskiego 1K, 15-245 Bialystok, Poland

**Keywords:** Chromium(VI), Mobility of Cr(III) and Cr(VI), Interconversion of chromium species, ETAAS, Environmental analysis

## Abstract

A critical appraisal of single-step extraction procedures of chromium species from soil was done in terms of their selectivity towards Cr(III) and Cr(VI) species. Samples of natural mineral and organic soil and samples of soil enriched with different chromium compounds of various solubility (in liquid or solid form) were used to simulate contamination of soil by liquid and solid wastes. The efficiency of extraction of Cr(III) and Cr(VI) species with various reagents, e.g. acetic acid, chelating agents (EDTA, DTPA) or inorganic salts (phosphates and carbonates), was evaluated on the basis of recovery results obtained for enriched samples. None of used reagents allow for quantitative extraction of added Cr(III) form. Procedures based on extraction of soil with Na_2_CO_3_ at room and elevated temperature (90–95 °C) were suitable for extraction of Cr(VI) species from mineral soil, whereas for organic soil, the procedure based on extraction with Na_2_CO_3_ at room temperature was recommended. The developed extraction procedures were validated using certified reference material (CRM 041 soil) and applied for analysis of contaminated soil samples. The studies showed that the physical state of waste, initial form and oxidation state of chromium and soil properties influenced the final chromium species and their mobility in soil, which have an impact on contamination of environment. The analysis of contaminated soil samples from a tannery area showed that the share of Cr(VI) was very low (only 0.8–4.5%) despite the high total content of chromium, which confirmed that chromium was present in immobile forms.

## Introduction

Chromium occurs in the environment mostly in two species, i.e. as a trivalent (III) and hexavalent (VI) form. Chromium has been proposed as an essential element; however, recent studies suggest that it should be removed from the list of essential trace elements. It was shown that Cr(III) plays a key role in chromium allergy and causes DNA damage in cell-culture systems (Vincent 2010). Cr(VI) (chromate) is known to be a genotoxic carcinogen due to the redox reactions that take place in cells which generate Cr(V)-1,2-diolato species (Chellan and Sadler 2015).

Chromium can enter the environment both from natural and anthropogenic sources, e.g. via electroplating, leather tanning and the textile industries (Avudainayagam et al. 2003; Johnson et al. 2006; Unceta et al. 2010; Dhal et al. 2013). Anthropogenic emission of chromium to the atmosphere is substantial and was estimated at 336 t in the European Union in 2013 (EEA Technical report 2015), 2700–2900 t in the USA and 21,000 t in China in 2009 (Cheng et al. 2014). Chromium released into the atmosphere is carried in the air as particles or dust. It may be transported over long distances by the wind, but finally settles on the soil. Rain will also remove chromium particles from the atmosphere and deposit them in the ground, thus contaminating the soil. The disposal of chromium-containing commercial products (e.g. some inks, paints and paper, rubber and composite floor coverings or toner powders used in copying machines) and coal fly ash from electric utilities and other industries are major sources of chromium releases into the soil (Barceloux 1999; Metze et al. 2005; Dhal et al. 2013). Solid waste and slag produced during chromate manufacturing processes as well as agricultural and food wastes, when disposed of improperly in landfills, can be another potential source of chromium exposure (Kimbrough et al. 1999; Barceloux 1999).

The Canadian Soil Quality Guidelines for the protection of human health recommend a maximum content for hexavalent chromium and total chromium in agricultural and residential land as 0.4 and 64 mg kg^−1^, respectively (Canadian Environmental Quality Guidelines 2001). The Swedish Guidelines (Guidelines for Polluted Soils 2002) suggest maximum concentrations for the most sensitive type of land use at 5 and 120 mg kg^−1^ for Cr(VI) and Cr(III), respectively. In Italy, the highest permissible Cr(VI) concentrations in soil are 2 and 15 mg kg^−1^, respectively, depending on the type of exploitation, i.e. parkland or industrial (Decreto Ministeriale n.471 1999; Pettine and Capri 2005a). In Poland, the limit for total chromium in agricultural and residential land was set at 150 mg kg^−1^ (Ordinance of the Minister of Environment of Poland 2016). The content of Cr_Tot_ and Cr(VI) in contaminated soil can reach a level of several grammes per·kilogramme (Dhal et al. 2013).

The behaviour of metals in soil and uptake by plants is controlled by element speciation and by soil properties, such as pH, particle size, cation-exchange capacity, content of organic matter, content and type of clay minerals and Al, Fe and Mn oxides, redox potential and microbiological activity (Kotaś and Stasicka 2000; Dhal et al. 2013; Krasnodębska-Ostręga et al. 2009; Paldyna et al. 2013). Chromium(III) in soil is mostly present as insoluble chromium(III) hydroxide and tends to be adsorbed on the soil surface in a pH range of 4–8. The solubility of Cr(III) in soil and its mobility may increase due to the formation of soluble complexes with organic matter in soil, e.g. citric acid, diethylenetriaminepentaacetic acid (DTPA) and fulvic acid. Hence, a lower soil pH potentially facilitates complexation (Avudainayagam et al. 2003; Kotaś and Stasicka 2000). The most mobile forms of Cr(VI) in soil are CrO_4_^2−^ and HCrO_4_^−^ ions, but insoluble species such as BaCrO_4_ and PbCrO_4_ may also be present (Kotaś and Stasicka 2000). Oxidation and reduction of chromium species in soil can take place simultaneously. Cr(VI) may react with many inorganic reductants such as Fe(II) and sulphide as well as with a number of organic compounds, including carboxylic and hydroxycarboxylic acids, aldehydes, phenols or fulvic acid (Eckert et al. 1990; Brose and James 2010). Moreover, several microorganisms possess the ability to reduce Cr(VI) (Brose and James 2010; Dhal et al. 2013). In soil containing manganese oxides, Cr(III) can be oxidised to Cr(VI), especially at high pH values (Dhal et al. 2013).

Various extraction procedures have been proposed in order to determine chromium(VI) species in solid environmental samples. The extraction conditions have to be carefully chosen as the leaching process may lead to interconversion of Cr species. The extraction solutions can be divided into the following groups: (a) acids at various concentrations, e.g. 0.43 mol L^−1^ acetic acid (CH_3_COOH) (Lillengen and Wibetoe 2002), (b) buffered salts, e.g. ammonium acetate (NH_4_OAc) (Morales-Muñoz et al. 2004) and K_2_HPO_4_ (Rüdel and Terytze 1999; James et al. 1995), (c) neutral salts such as CaCl_2_ (Béni et al. 2007), Na_3_PO_4_ (Mandiwana 2008) or Na_2_CO_3_ (Panichev et al. 2003; Elci et al. 2010), (d) chelating agents, e.g. ethylenediaminetetraacetic acid (EDTA) (Lillengen and Wibetoe 2002; Korolczuk and Grabarczyk 2005), DTPA (Grabarczyk et al. 2006) and S,S-ethylenediamine-N,N′-trisodium salt (EDDS) (Grabarczyk 2008) and (e) other extractants (Gitet et al. 2013) proposed for routine soil testing. Some of these procedures have been designed in order to distinguish between soluble, exchangeable and slightly soluble forms of chromium(VI).

Alkaline media have been suggested for selective extraction of slightly soluble Cr(VI), as in such an environment, Cr(VI) is stable in the solution while Cr(III) species form insoluble hydroxides or carbonates. Insoluble forms of Cr(VI) are often extracted with suitable chelating agents by forming soluble complexes of cations. At the same time, Cr(VI) is transferred into the solution as a soluble salt. The most recommended method for determining Cr(VI) in solid matrices is the US EPA method 3060A, which was designed and validated by Vitale et al. (1994, 1995). In this method, a hot (90–95 °C) 0.28 mol L^−1^ Na_2_CO_3_ solution in 0.5 mol L^−1^ NaOH is used to extract the “total” amount of Cr(VI) from the soil and sediments. However, the presence of the reducing compounds results in an underestimated concentration of Cr(VI) in the extraction solution (Malherbe et al. 2011). Conversely, some authors have observed partial oxidation of soluble Cr(III) and a positive error in Cr(VI) determination (Huo et al. 1998; Huo and Kingston 2000). A brief description of these procedures, their effectiveness and application for solid samples is presented in Table 1.

**Table 1:** Extraction procedures of Cr(VI) from solid samples

Extracted forms	Extraction solution	Extraction conditions		Recovery of Cr(VI), %	Analysed matrices	Reference
		*m*:*V*	Time			
Water-soluble	Deionised water	1:100	24 h shaking	0.07^a^	Soil	Elci et al. 2010
Naturally extracted	Deionised water, CO_2_	1:100	24 h shaking	0.06^a^	Soil	Panichev et al. 2003
Soluble	0.43 mol L^−1^ CH_3_COOH	1:50	16 h shaking	0.66	CRM 483—sewage sludge-amended soil	Lillengen and Wibetoe 2002
Soluble	0.04 mol L^−1^ (NH_4_)_2_SO_4_, 0.5 mol L^−1^ NH_4_OH (pH ~8)	1:10	14 min, 300 W (microwave extraction)	93 112.6	River sediment + 50 μg g^−1^ Cr(VI) as K_2_CrO_4_ River sediment + 30 μg g^−1^ Cr_2_O_3_	Morales-Muñoz et al. 2004
Soluble	0.12 mol L^−1^ K_2_HPO_4_ (pH ~8.2), 0.37 mol L^−1^ Al_2_(SO_4_)_3_, 0.94 mol L^−1^ Na_2_SO_3_	1:5	30 min shaking	12 88	Soil + 10 μg g^−1^ Cr(VI) as PbCrO_4_ Soil + 12.5 μg g^−1^ Cr(VI) as K_2_Cr_2_O_7_	Rüdel and Terytze 1999
Soluble and exchangeable	0.1 mol L^−1^ KH_2_PO_4_-K_2_HPO_4_ (pH ~7)	1:10	16 h shaking	29	Soil + 10–20 mg Cr(VI) as BaCrO_4_	James et al. 1995
Soluble and insoluble	0.01 mol L^−1^ Na_3_PO_4_ (pH ~12)	1:100	5 min boiling	98	CRM 027—sandy loam	Mandiwana 2008
Soluble and insoluble	0.1 mol L^−1^ Na_2_CO_3_ (pH ~10)	1:100	10 min boiling	98	CRM 545—welding dust	Elci et al. 2010
Soluble and insoluble	0.28 mol L^−1^ Na_2_CO_3_, 0.5 mol L^−1^ NaOH (pH ~12), 4 mol L^−1^ MgCl_2_, 1 mol L^−1^ K_2_HPO_4_-KH_2_PO_4_ (EPA 3060A)	1:20	60 min heating at 90–95 °C	95	Soil spiked with Cr(VI)	Gitet et al. 2013
Soluble and insoluble	0.5 mol L^−1^ NaOH, 0.28 mol L^−1^ Na_2_CO_3_	1:20	60 min heating at 90–95 °C	88 100 63	CRM 013—paint chips CRM 013 + 2600 μg g^−1^ Cr(III) as CrCl_3_ Soil + 10–20 mg Cr(VI) as BaCrO_4_	Korolczuk and Grabarczyk 2005
Soluble and insoluble	0.05 mol L^−1^ EDTA	1:50	60 min shaking	1 0.91	CRM 483—sewage sludge-amended soil CRM 07411 Chinese soil	Lilleengen and Wibetoe 2002
Soluble and insoluble	0.01 mol L^−1^ EDTA in 0.05 mol L^−1^ NH_4_OH + (NH_4_)_2_SO_4_ (pH ~9.5)	1:100	30 min, 40 °C (ultrasound-assisted extraction)	96.5–98.4 86	SiO_2_ spiked with 400 μg g^−1^ Cr(VI) as insoluble chromate CRM 013—paint chips	Korolczuk and Grabarczyk 2005
Soluble and insoluble	0.02 mol L^−1^ DTPA in 0.05 mol L^−1^ NH_4_OH + (NH_4_)_2_SO_4_ (pH ~9.5)	1:50	10 min heating at 40 °C, stirring	97	SiO_2_ spiked with 1.125 μg g^−1^ Cr(VI) as BaCrO_4_ and SrCrO_4_	Grabarczyk et al. 2006

*m* mass of sample, *V* volume of extraction solution

CRM 483—sewage sludge-amended soil (Cr content 3925 ± 195 μg g^−1^); CRM 027—sandy loam (Cr content 26.9 ± 0.9 μg g^−1^); CRM 545—welding dust loaded on filter (Cr(VI) content 40,200 ± 0.6 μg g^−1^); CRM 07411—Chinese soil (Cr content 61.8 ± 2.1 μg g^−1^); CRM 013—paint chips (Cr content 618 μg g^−1^)

^a^Efficiency of Cr extraction

It should be noted that the extraction procedures are not always selective for Cr(VI), as differentiation between oxidised and reduced Cr species may be obtained by using specific analytical methods, e.g. the diphenylcarbazide (DPC) method for Cr(VI). However, many papers have indicated interference in spectrophotometric chromium(VI) detection caused by the presence of other metal ions, e.g. Cu(II), Mo(VI), Fe(III), V(V) and Hg(II), and humic acids released from the soil (Pettine and Capri 2005b). Determining the chromium via a specific spectroanalytic detection technique, e.g. atomic absorption spectrometry, provides more accurate results and a lower detection limit. However, in this case, selectivity of the extraction procedures towards Cr(VI) and Cr(III) species should be assessed.

The aim of this work was to appraise the procedures originally proposed for the leaching of chromium(VI) species from solid samples in terms of their selectivity towards trivalent and hexavalent chromium species. For this reason, mineral and organic soil was spiked with soluble and slightly soluble chromium compounds (CrCl_3_ 6H_2_O, K_2_Cr_2_O_7_, BaCrO_4_). The spikes were dosed in liquid and solid forms in order to evaluate the mobility of chromium from waste disposal sites. The recoveries of chromium obtained after treating the soil with seven extraction procedures were controlled by electrothermal atomic absorption spectrometry (ETAAS) and compared in order to select the most suitable procedure for extraction of Cr(VI) and Cr(III) species. The trueness of the procedure proposed for selective extraction of Cr(VI) was verified in an analysis of certified reference material of soil (CRM 041). The chosen extraction procedures were applied to determine Cr(VI) in soils sampled in the Podlasie Province (Poland).

## Material and Methods

### Instrumentation

A Solaar M6 (Thermo Electron Corporation, UK) atomic absorption spectrometer equipped with a Zeeman-effect background correction system and an electrothermal atomizer (ELC graphite tubes) was used for chromium determination. A chromium hollow cathode lamp (Photron, Australia) was operated at 15 mA. The measurements were done at *λ* = 357.9 nm with a spectral bandpass of 0.5 nm. The following optimised heating programme was used to determine chromium in the soil extracts: drying at 110 °C for 15 s, ashing at 1200 °C or 1650 °C for 8 s, and atomisation at 2600 °C for 3 s. A temperature of 1650 °C was used to ash extracts containing K_2_HPO_4_ in the presence of Mg(NO_3_)_2_ (10 μL of 10 μg mL^−1^, 0.1 μg) as a chemical modifier. An inoLab pH Level 1 (WTW, Germany) pH meter equipped with a SenTix 21 electrode (WTW, Germany) was used for the pH measurements. A Shimadzu SSM-5000A TOC Analyser was used to determine the carbon content in the soil by catalytically aided combustion oxidation method. A ball mill (KM 1 type K142, MLW, Poland) was used for soil grinding.

### Reagents

Stock solutions (20 g L^−1^) of Cr(III) as CrCl_3_ (Merck, Germany) and (1.001 g L^−1^) of Cr(VI) as K_2_Cr_2_O_7_ (Sigma-Aldrich, Germany) were used. Working standard solutions of chromium were prepared daily by appropriate dilution of the stock standards. Reagents used for chromium extraction: K_2_HPO_4_, Na_3_PO_4_, Na_2_CO_3_ and EDTA as well as reagents used for spiking of soil CrCl_3_ 6H_2_O, K_2_Cr_2_O_7_ and BaCrO_4_ were obtained from POCh (Poland). Acetic acid and KCl were obtained from Chempur (Poland), and DTPA was obtained from Sigma-Aldrich (Germany). Deionised water was obtained from the Milli-Q water purification system (Millipore, USA).

### Samples and Procedures

#### Model Soils

Two different soils, i.e. in terms of the physico-chemical properties (agricultural type, pH, content of the organic matrix), were collected from the arable layer in the Podlasie Province (Poland). The soil was air-dried, homogenised and sieved using a 1-mm sieve. Both the content of organic carbon and the pH of the soils (in KCl) were determined by using standard methods. The mineral soil (M) contained 1.6% organic carbon (pH_KCl_ = 5.4), while the organic soil (O) contained 10.5% organic carbon (pH_KCl_ = 6.1). The samples were spiked with different chromium compounds and used for optimisation of the extraction procedure.

#### Spiking Procedures

Three portions of each soil (5 g) were spiked with 50 mg g^−1^ of Cr(III) or Cr(VI) in a solid form (CrCl_3_·6H_2_O, K_2_Cr_2_O_7_, BaCrO_4_). The samples were homogenised for 1 h in a ball mill. Next, 0.25 g of spiked soil was mixed with 4.75 g of natural soil and the sample was homogenised again for 1 h. One gramme of each soil was mixed with 49 g of natural soil for 16 h. In effect, each portion of soil (50 g) was spiked with 50 μg g^−1^ of chromium as CrCl_3_ 6H_2_O (O-Cr(III)s, M-Cr(III)s), K_2_Cr_2_O_7_ (O-Cr(VI)s, M-Cr(VI)s) and BaCrO_4_ (O-Cr(VI)s*, M-Cr(VI)s*). Two other portions of soil (50 g) were shaken with 50 mL of 50 μg mL^−1^ solution of Cr(III) or Cr(VI) compounds, such as CrCl_3_ 6H_2_O (O-Cr(III)_liq_, M-Cr(III)_liq_) and K_2_Cr_2_O_7_ (O-Cr(VI)_liq_, M-Cr(VI)_liq_)), for 2 h and dried in air. These samples were then used for further studies.

#### Determination of the Total Content of Cr

The content of total chromium in both the natural and spiked soils was determined after wet mineralisation of soil in a mixture of HNO_3_:HF (5 mL:1 mL). Samples (0.2 g) were heated in closed Teflon vessels in a microwave digestion system (Ethos Plus, Milestone, Italy) according to optimised microwave program: 250 W for 2.5 min, 500 W for 5 min and 700 W for 15 min. The process was repeated twice for total digestion of soil. The obtained solutions were transferred into polyethylene vessels, diluted with Milli-Q water to the final volume of 15 mL and analysed by ETAAS. The chromium content in the mineral soil (M) was 16.9 μg g^−1^, while in the organic soil (O) it was 18.3 μg g^−1^. The average content of chromium in mineral soil spiked with different chromium compounds was 34.2 ± 5.1 μg g^−1^ (the average spiking efficiency was equal to 68%), while in the spiked organic soil it was 38.4 ± 4.5 μg g^−1^ (the average spiking efficiency was equal to 76%).

#### Soil Samples

The soils examined in this work were collected from industrially contaminated area of a closed down leather tannery in Krynki (Podlasie Province, Poland). The content of Cr(VI) in soil was evaluated by using the developed procedure.

#### CRM

Certified reference material of sandy clay soil CRM 041 (Sigma-Aldrich, Germany) with certified Cr(VI) content (86.3 ± 2.96 μg g^−1^) was used for the accuracy studies.

#### Extraction Procedures

The extraction procedures used to evaluate the efficiency of chromium released from the soils are summarised in Table 2. Uniform extraction conditions were always used. A total of 50 mL of one of the extraction solutions was added to each soil sample (mass of 1 g). The suspensions were rotated for 16 h at room temperature (22 ± 2 °C) and next centrifuged at 3000 rpm for 15 min. The tubes for the hot carbonate extraction, after the addition of extraction solution, were placed on a preheated hot plate and maintained at 90–95 °C for 10 min. The supernatants, after centrifugation and appropriate dilution (5–100 times), were used to determine the chromium concentration by ETAAS. All analyses were carried out in triplicates. Recovery of spiked chromium obtained in various extraction procedures was assessed as follows:

$$ R\left(\%\right)=\left({m}_{\mathrm{exsp}}{\textstyle \hbox{-} }{m}_{\mathrm{exnat}}\right)/{m}_{\mathrm{sp}}\times 100\% $$

where *m*_exsp_—mass of Cr extracted from spiked soil

*m*_exnat_—mass of Cr extracted from soil

*m*_sp_—mass of spiked Cr

The mass of chromium extracted from soil was calculated on the basis of an external calibration graph prepared for the given extracting solution.

**Table 2:** The efficiency of Cr extraction from natural soil and the recovery of Cr(III) and Cr(VI) from spiked soil by using various extraction solutions (mass of soil 1 g, volume of extraction solution 50 mL, extraction for 16 h at room temperature)

Extraction solution	Type of soil	Efficiency of Cr extraction, %	Recovery of Cr, %	
		Natural soil	Soil spiked with Cr(III)	Soil spiked with Cr(VI)
0.43 mol L^−1^ CH_3_COOH (pH ~2.7)	Organic	0.28	1.9–13.1	14.6–23.2
	Mineral	0.25	1.7–16.0	14.9–29.2
0.1 mol L^−1^ K_2_HPO_4_ with addition of 1 mL 0.37 mol L^−1^ Al_2_(SO_4_)_3_ + 1 mL 0.94 mol L^−1^ Na_2_SO_3_ (pH ~8.0; 1.56 mol L^−1^ H_3_PO_4_)	Organic	1.0	0.7–7.4	4.6–14.1
	Mineral	0.4	0.9–1.4	5.5–12.1
0.01 mol L^−1^ Na_3_PO_4_ (pH ~11.0)	Organic	4.7	1.4–15.8	16.6–80.1
	Mineral	4.3	2.1–17.0	34.7–77.9
0.1 mol L^−1^ Na_2_CO_3_ (pH ~10.0)	Organic	10.7	2.0–35.6	35.6–109.8
	Mineral	1.7	1.5–8.1	34.7–111.5
^a^0.1 mol L^−1^ Na_2_CO_3_ (pH ~10.0)	Organic	14.2	5.3–73	68.7–98.2
	Mineral	6.0	3.5–22.9	43.2–99.8
0.01 mol L^−1^ EDTA (pH ~9.5; 0.05 mol L^−1^ (NH_4_)_2_SO_4_ + 25% NH_4_OH)	Organic	7.7	3.2–37.5	42.9–106.4
	Mineral	3.3	2.0–17.2	53.9–110.7
0.02 mol L^−1^ DTPA (pH ~9.5; 0.05 mol L^−1^ (NH_4_)_2_SO_4_ + 25% NH_4_OH)	Organic	12.8	3.8–48.3	34.0–98.9
	Mineral	3.5	10.5–50.4	51.2–103.4

^a^ extraction by heating of suspension at 90–95 °C for 10 min

## Results and Discussion

### An Appraisal of Extraction Procedures of Chromium Species from Soil

**Fig. 1:**
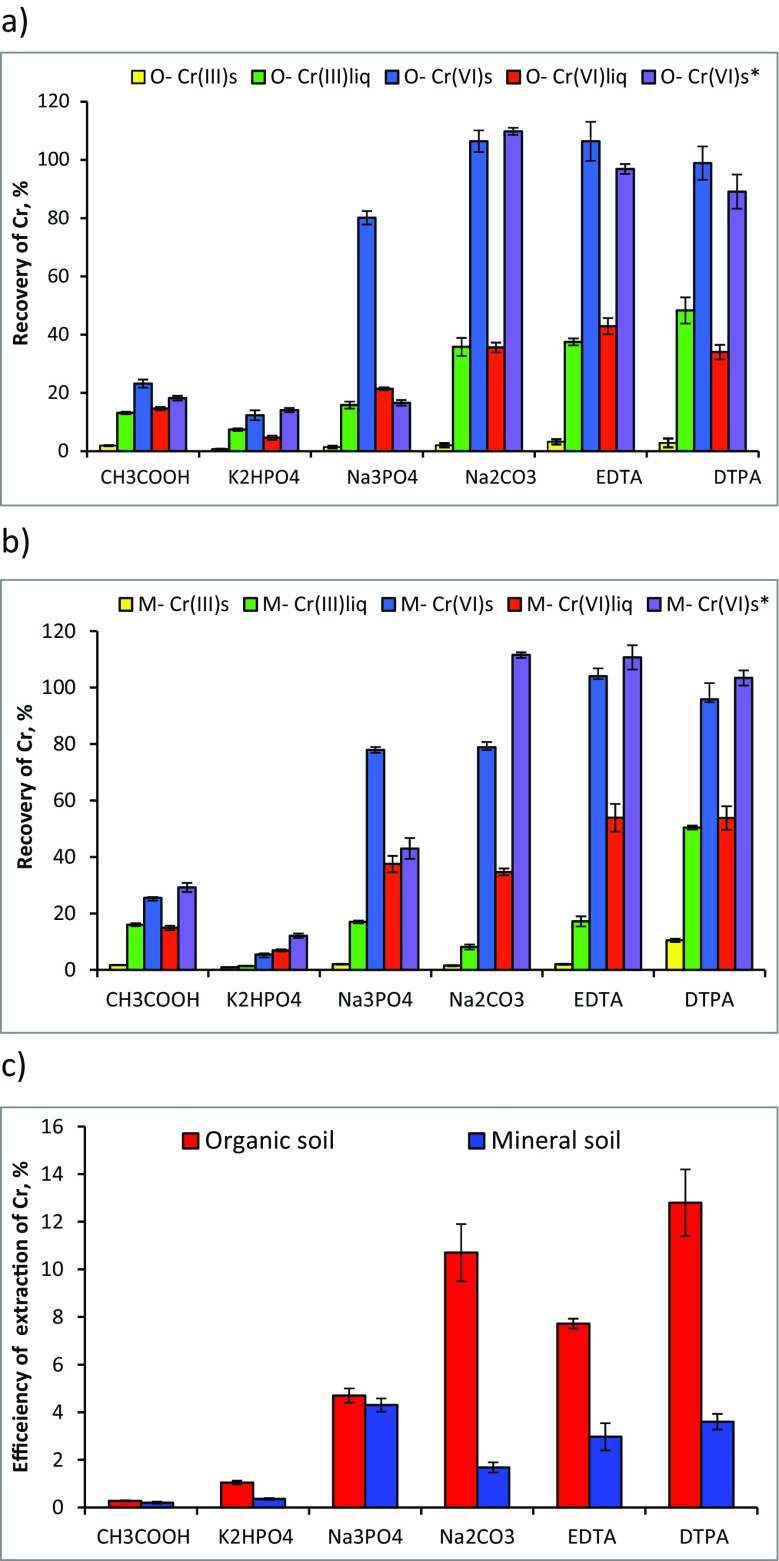
The recovery of chromium from soil spiked with Cr(III) and Cr(VI) in solid (s) or liquid (liq) forms extracted with using various reagents. **a** Organic soil. **b** Mineral soil. **c** The efficiency of chromium extraction from native soil with using various reagents (value ± standard deviation, *n* = 3)

The selectivity of procedures originally proposed for leaching of chromium(VI) species from solid samples (listed in Table 1) was tested. The mass of chromium extracted from mineral and organic soil spiked with different trivalent and hexavalent compounds of chromium (as CrCl_3_·6H_2_O, K_2_CrO_4_, BaCrO_4_) was determined, and recoveries were calculated (Table 2). The obtained results are shown in Fig. 1. For a comparison, all procedures were also applied for extraction of native chromium from unpolluted soils. The efficiency of chromium extraction was higher from organic soil (0.3–13% of its total content) than from mineral soil (0.3 to 4.4%) (Fig. 1c).

Acetic acid at various concentrations was recommended in order to determine the exchangeable and carbonate-bound chromium fraction in the soil (Lillengen and Wibetoe 2002; Krasnodębska-Ostręga et al. 2009). The amount of native chromium extracted from natural soil with 0.43 mol L^−1^ solution of CH_3_COOH was very low (<0.3%). The recovery of chromium from soils spiked with Cr(VI) forms was higher (14–29%) than from soils spiked with Cr(III) forms (1.7–16%). It was found that Cr(III) added to the soil in a liquid form was extracted in a higher amount (13–16%) than that added in a solid form (1.7–1.9%). The opposite correlation was observed for Cr(VI) (Fig. 1a, b).

Some papers mentioned that extraction of Cr(VI) in alkaline solution is preferable to acid extraction because it ensures better solubility of some of the chromate compounds (James et al. 1995). The extraction procedure using phosphate buffer is devoted to the leaching of soluble Cr(VI) forms (Rüdel and Terytze 1999). The addition of Al_2_(SO_4_)_3_ enhances precipitation of Cr(III) in the sulphate form, while Na_2_SO_3_ prevents oxidation reactions. The efficiency of chromium extraction from natural soil with 0.1 mol L^−1^ solution of K_2_HPO_4_ was slightly higher (0.4–1%) than that with CH_3_COOH. However, this procedure was less effective for the extraction of chromium(VI) from spiked soils as its recovery was in the range of 4.6–14.1%; surprisingly, it was lower for the soluble (as K_2_CrO_4_) than for the insoluble (as BaCrO_4_) form of Cr(VI) (Fig. 1a, b). Such low recovery of insoluble Cr(VI) from the soil (<12%) was also reported by others (Szulczewski et al. 1997). This procedure is then not recommended for the extraction of total Cr(VI), as was proposed in James et al. (1995). It is worth noting that up to 7.4% of spiked Cr(III) was also extracted with this reagent.

During extraction of soil with the Na_3_PO_4_ solution, insoluble Cr(VI) compounds (e.g. BaCrO_4_) are transformed into soluble forms (e.g. Na_2_CrO_4_) (Mandiwana 2008). The procedure should remove all common metals, e.g. insoluble phosphates, oxides or hydroxides, so the solution should contain only CrO_4_^2−^ ions. The efficiency of chromium extraction from natural soil with 0.01 mol L^−1^ Na_3_PO_4_ was below 5%. Higher recovery of chromium was obtained from soil spiked with solid K_2_CrO_4_ (80%) than from soil spiked with BaCrO_4_ (20–40%). Recovery of Cr(III) forms was similar to that obtained with CH_3_COOH, namely 2% from soil spiked with solid CrCl_3_ and 17% from soil spiked with CrCl_3_ solution (Fig. 1a, b).

**Fig. 2:**
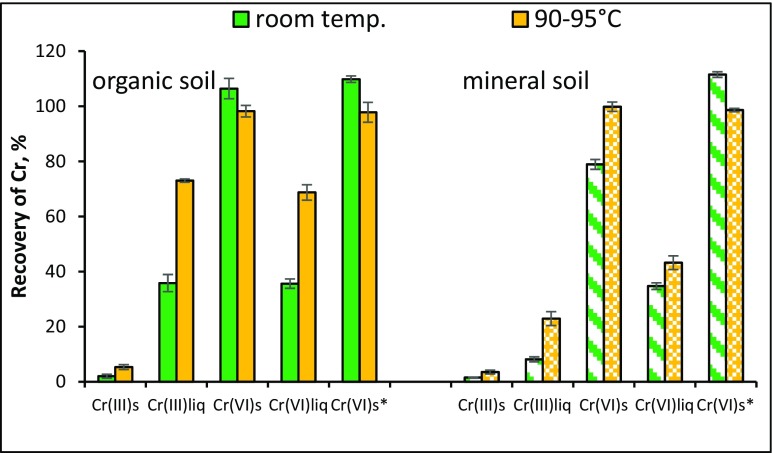
The recovery of chromium from soil spiked with Cr(III) and Cr(VI) in solid (s) or liquid (liq) forms extracted with Na_2_CO_3_ solution at room temperature or heated to 90–95 °C (value ± standard deviation, *n* = 3)

Leaching of soil with 0.1 mol L^−1^ Na_2_CO_3_ in alkaline solution was proposed for extraction of soluble and insoluble Cr(VI) forms (Vitale et al. 1994). Such treatment of samples transformed insoluble Cr(VI) salts into soluble chromate ions, whereas all common metals, including Cr(III), were removed as insoluble carbonates, oxides or hydroxycarbonates. The efficiency of extraction of native Cr with this reagent was 2% from mineral and 11% from organic soil. The recovery of chromium added as a solid Cr(III) salt was about 2% from both types of soil, while added as a solution, it increased to 8 and 35% from mineral and organic soil, respectively. High recovery of chromium (80–110%) from both types of soil spiked with soluble and insoluble solid Cr(VI) salts was observed. Lower recovery of chromium added as a solution of CrO_4_^2−^ suggests that Cr(VI) was partly reduced by the sample matrix to insoluble Cr(III), which supports the observations of Vitale et al. (1997) that soluble CrO_4_^2−^ is reduced to insoluble Cr_2_O_3_. This effect was also observed in all other procedures (Fig. 1a, b). In order to shorten the procedure, the extraction of soil with Na_2_CO_3_ solution at a high temperature, i.e. 90–95 °C, for 10 min (Panichev et al. 2003) was tested for Cr(VI) leaching. It was observed that the colour of the alkaline extract was much darker, indicating that at higher temperature, the organic components of soil, e.g. humic acids, were better solubilised. This effect was more visible for organic soil. A small increase was observed in the extraction efficiency of native chromium (~4%) and the recovery of solid Cr(III) spike (~2%) from organic and mineral soil. The recovery of Cr(VI) spikes added as a solid in soluble and insoluble forms from both types of soil was quantitative, which indicates that the presence of liberated organic compounds in the extract did not significantly influence the reduction process of Cr(VI) species (Vitale et al. 1997). The highest increase in the recovery of analytes (~35%) in comparison to unheated treatment was observed for organic soil spiked with solutions of Cr(III) and soluble Cr(VI) (Fig. 2). The increase in recoveries of analytes from mineral soil spiked with the same chromium forms was slightly lower, i.e. 15% for Cr(III) and 8% for soluble Cr(VI). The observed changes suggest that immobile chromium species were converted into mobile forms. Most probably under alkaline conditions, high temperature and the presence of carbonate and organic matter, Cr(III) was oxidised to Cr(VI), which was extracted more efficiently. Extraction in high temperature resulted in the release of chromium species adsorbed on soil particles due to the dissolution of the organic and inorganic substances present in the soil. The studies showed only a small increase in the extraction efficiency of chromium from native soil (by 4%) at an elevated temperature. This suggests that native chromium is present in a form that is more resistant to oxidation and solubilisation.

Extraction of chromium with EDTA and DTPA solutions is based on the transformation of insoluble Cr(VI) compounds into soluble species by complexation of the metal ions that form insoluble chromates (Korolczuk and Grabarczyk 2005; Grabarczyk et al. 2006). The extraction of chromium with EDTA and DTPA was similar to that obtained with the Na_2_CO_3_ solution. The efficiency of extraction of chromium from natural soil was in the range of 3.3–12.8%. Chromium(VI) added in a solid form was quantitatively recovered from both soils. The recoveries of Cr(III) and Cr(VI) spiked as a liquid were in the range of 17–54%. A higher percentage of metal was extracted with EDTA than with DTPA from soil spiked with the Cr(VI) form. The results showed that EDTA and DTPA were the most efficient extractants of Cr(VI) among all the tested reagents; however, they also leached significant amounts of Cr(III) (Fig. 1a, b).

On the basis of the performed experiments, the tested reagents were sorted according to their extraction potential into Cr(III) and Cr(VI) species. For the Cr(III) species, the differences in the extraction power of the reagents used were observed depending on the phase of the introduced spike. For Cr(III) added as a solid to mineral and organic soil, the order of extraction reagents was as follows: K_2_HPO_4_ (~1%) < CH_3_COOH, Na_3_PO_4_, Na_2_CO_3_ (~2%) ≤ EDTA (2–3.2%) < DTPA (3.8–10%). When Cr(III) was introduced as a liquid, a different extraction order of reagents was observed for the two tested types of soil. The spike of Cr(III) from mineral soil was extracted with increasing efficiency by using the following reagents: K_2_HPO_4_ (1.4%) < Na_2_CO_3_ (8%) < CH_3_COOH, Na_3_PO_4_, EDTA (~17%) < DTPA (50%), whereas from organic soil the order was as follows: K_2_HPO_4_ (7%) < CH_3_COOH, Na_3_PO_4_ (~15%) < Na_2_CO_3_, EDTA (~36%) < DTPA (48%). For organic soil, the extraction of Cr(III) with hot Na_2_CO_3_ was the most efficient procedure (recovery: 5.3% for solid spike and 73% for liquid spike). For mineral soil, this procedure had a higher extraction power than CH_3_COOH, Na_3_PO_4_ and EDTA, but still lower than DTPA (recovery: 3.5% for solid spike and 22% for liquid spike). It should be noted that none of the tested procedures allowed for total recovery of Cr(III), which indicates a strong interaction of the spike with the matrix of the soil. The highest recovery of Cr(III) from both types of soil was obtained with DTPA solution (~50%).

Spikes of Cr(VI) introduced into the organic soil in either a liquid or solid phase were extracted with increasing efficiency by using the following reagents: K_2_HPO_4_ (5–14%) < CH_3_COOH (15–23%) < Na_3_PO_4_ (17–80%) < DTPA, Na_2_CO_3_, EDTA (35–110%). The order of reagents for mineral soil spiked with insoluble Cr(VI) was the same as for organic soil. In the case of mineral soil spiked with soluble Cr(VI) (as a liquid and solid form), similar power of Na_3_PO_4_ and Na_2_CO_3_ towards Cr(VI) was observed; therefore, the order of the reagents was slightly different: K_2_HPO_4_ (5–7%) < CH_3_COOH (15–25%) < Na_3_PO_4_ = Na_2_CO_3_ (35–78%) < DTPA, EDTA (51–105%). The total recovery of solid Cr(VI) spikes from organic soil was obtained using Na_2_CO_3_, DTPA and EDTA solutions, whereas from mineral soil it was obtained with DTPA and EDTA solutions. The most efficient extraction of Cr(VI) from both samples was obtained with hot Na_2_CO_3_, thus allowing for quantitative recovery of the solid Cr(VI) spike.

The order of the tested reagents towards extraction of native chromium from organic soil was the same as for the spike of Cr(VI) and was as follows: CH_3_COOH (0.3%) < K_2_HPO_4_ (1%) < Na_3_PO_4_ (4.7%) < EDTA (7.7%) < Na_2_CO_3_ (11%) < DTPA (12.8%) < Na_2_CO_3_ hot (14.2%). For mineral soil, higher extraction power towards native chromium was demonstrated by the Na_3_PO_4_ solution and hot Na_2_CO_3_; therefore, the order of extraction reagents was as follows: K_2_HPO_4_, CH_3_COOH (0.3%) < Na_2_CO_3_ (1.6%) < EDTA, DTPA (3.5%) < Na_3_PO_4_ (4.4%) < Na_2_CO_3_ hot (6%).

It was found that the CH_3_COOH and K_2_HPO_4_ solutions exhibited the lowest extraction power towards all spikes of chromium, which is consistent with the literature data suggesting that these reagents be used for extraction of soluble Cr(VI) forms (Lillengen and Wibetoe 2002; Rüdel and Terytze 1999). Lower recovery of Cr(VI) spiked as a soluble chromate was obtained with K_2_HPO_4_ than was reported in Rüdel and Terytze (1999) (~12 versus 88%), whereas a similar recovery was obtained for insoluble chromates (~12%). Among the other reagents proposed for extraction of soluble and insoluble Cr(VI) forms, only Na_3_PO_4_ was ineffective, even when applied by others (Mandiwana 2008). The results obtained in our experiments in procedures using Na_2_CO_3_, EDTA and DTPA solutions were comparable to the results presented in the literature for samples spiked with Cr(VI) in the form of soluble and insoluble chromate (see Table 1) (Korolczuk and Grabarczyk 2005; Grabarczyk et al. 2006; Lillengen and Wibetoe 2002). The efficiency of extraction of native chromium with CH_3_COOH and EDTA as obtained in our work was the same as that reported by Lillengen and Wibetoe (2002) for CRM 483 sewage sludge-amended soil and CRM 07411 Chinese soil.

The choice of procedure for selective extraction of Cr(VI) using 0.1 mol L^−1^ Na_2_CO_3_ at room temperature is highly recommended for mineral soil samples, as it provided quantitative recovery of Cr(VI) and slight extraction of Cr(III) (8%). This procedure may also be appropriate for natural organic soil, despite the higher recovery of Cr(III), which was probably the effect of better solubility of the spiked form of chromium (CrCl_3_). Taking into account the efficiency of extraction of native chromium from both mineral and organic soil, in which Cr(III) forms predominate, and the advantage of shortening the extraction time, the extraction procedure using Na_2_CO_3_ at high temperature may also be recommended for mineral soil.

As was discussed above, releasing chromium from native (model soils) and anthropogenic contaminated soil (spiked soils) was different. Therefore, the characteristics of the environmental samples and the sources of their contamination should be known to properly select the extraction procedure.

### An Impact of Physical Form of Waste on Chromium Mobility in Soil

The studies performed here show that the differences in the recovery of spiked chromium forms depend not only on the chromium oxidation state and type of soil but also on the physical phase of the spike. Cr(III) forms, considered to be immobile and insoluble, were partly extracted from the soil, though less efficiently when Cr(III) was spiked in a solid form than in a liquid form. The recovery of Cr(III) added as a solid compound was only 4% from the organic soil and 2% from the mineral soil, irrespective of the extraction procedure that was used (Fig. 1a, b). Higher recovery of chromium with the DTPA solution (up to 10%) was observed, which might be an effect of the weak dissolution of the solid spike due to a shift in the chemical equilibrium towards complexation of Cr(III) ions with the chelating reagent. Such an effect was not observed for extraction with EDTA due to the slow rate of the formation of Cr(III)-EDTA complexes. Cr(III) added to the soil in the form of a solution was extracted to a higher extent. Its recovery from mineral soil reached 17%, while from organic soil it was 37%. Also in this case, the recovery of chromium with DTPA solution from both types of soil was highest (up to ~50%). Low recoveries of added Cr(III) ions suggest the occurrence of a strong interaction between the spike and the soil. Probably, Cr(III) cations, added as a spike, are immobilised/sorbed on the negatively charged surface of particles of the soil components. The precipitation of Cr(OH)_3_, which gradually undergoes dehydration and crystallisation as Cr_2_O_3_, is also possible. Higher recoveries of Cr(III) (35–48%) obtained from the organic soil by Na_2_CO_3_, EDTA and DTPA solutions suggest that some soluble organic complexes of Cr(III) with fulvic/humic or citric acid could also be formed in the presence of humic substances.

Since the phenomenon of inter-conversion of chromium oxidation forms under environmental conditions has already been reported (Kotaś and Stasicka 2000; Metze et al. 2005; Dhal et al. 2013), oxidation of the added Cr(III) to Cr(VI) in the tested soil could not be excluded. Such oxidation may arise only under certain circumstances in the presence of MnO_2_ or molecular oxygen (Apte et al. 2005; Pantsar-Kallio and Oksanen 2001). Dhal et al. (2013), in their review, stated that oxygen does not react appreciably with Cr(III), while oxidation of Cr(III) by MnO_2_ depends on the concentration of water-soluble chromium, pH of soil, amount of organic matter and drainage of the soil. For the studied samples, the presence of Cr(III) in the aqueous phase of soil was limited due to its adsorption on the soil particles, the precipitation and formation of stable complexes with humic substances. Therefore, oxidation of the added spike of Cr(III) during extraction at room temperature was less probable. However, during heating of soil with Na_2_CO_3_ solution, the probable oxidation of Cr(III) to mobile Cr(VI) forms arose, as higher recovery of Cr spikes was observed. The alkaline conditions, high temperature and presence of organic matter led to oxidation of Cr(III) to the Cr(VI) form.

The recoveries of Cr(VI) forms, which are considered to be more mobile than Cr(III) forms, were also dependent on the physical state of Cr(VI) added to the soil. Generally, Cr(VI) added as a solution was less extractable than when added as a solid compound (soluble and insoluble salts). Probably, such a phenomenon was the effect of the reduction of Cr(VI) to Cr(III) by the matrix components of the soil. Although the reduction of Cr(VI) to Cr(III) by the soil organic compounds and the oxidation of Cr(III) to Cr(VI) by the manganese oxides result from thermodynamically spontaneous reactions (James 2001), the reduction process arises more easily than oxidation under environmental conditions (Dhal et al. 2013; Brose and James 2010). The concentration of the reducing agents such as organic matter, sulphides or Fe(II)-bearing minerals in the soil and the soil pH affect the redox behaviour of chromium (Apte et al. 2005; Metze et al. 2005). The rate of reduction of Cr(VI) by the humic acids increases along with the decrease in pH (Dhal et al. 2013). The content of organic matter (18.1%) and acidic conditions in organic soil (pH 6.1) facilitated the reduction of the added Cr(VI) to Cr(III) to a large extent. In aerobic soil, which contains appropriate organic substances for the redox process, the reduction of Cr(VI) in a slightly alkaline environment is also possible. The formed Cr(III) species were probably immobilised on the soil particles. Therefore, in all of the tested extraction solutions, the recovery of Cr(VI) added as a solution to the organic soil was almost the same as the recovery of the liquid spike of Cr(III). For the mineral soil, the acidic conditions enhanced the rate of release of the Fe(II) species from the minerals, which could then react with Cr(VI) in the aqueous phase. However, higher recovery of Cr(VI) than Cr(III) (both added in the liquid form) indicates that a smaller amount of Cr(VI) was converted into Cr(III) in the mineral soil.

The obtained results demonstrate the transformation of added chromium forms in the soil, which leads to a significant change in their initial mobility and finally in the recovery of added forms of chromium. The most important conclusion from this experiment is that the mobility of chromium species depends not only on its oxidation state but also on its physical form as introduced into the environment, which is of great significance when chromium waste is disposed of into the environment.

### Validation of Extraction Procedures of Cr(VI) from Soil

Validation of the extraction procedures based on using 0.1 mol L^−1^ Na_2_CO_3_ solution, shaking the suspension of the sample for 16 h at room temperature or heating the suspension of the sample at 90–95 °C for 10 min was performed in order to obtain reliable and accurate results of chromium species extracted from the soil samples. During method validation, the following parameters were estimated: linearity, limit of detection (LOD) and limit of quantification (LOQ), precision, trueness and uncertainty of measurements of Cr(VI) content in soil extracted with Na_2_CO_3_.

In order to verify the linearity of the calibration graph, Cr(VI) standards in a concentration range of 1–50 ng mL^−1^ were prepared in a 100 times diluted Na_2_CO_3_ extraction solution and their absorbance was measured by ETAAS. Then the calibration graph was constructed as *A* = *f*(*C*_Cr(VI)_) and the correlation factor was used to verify their linearity. It was found that the correlation factor (*R*) was higher than 0.995 for the calibration graph prepared from the standard solutions at a concentration range of 1–30 ng mL^−1^. The obtained equation of the calibration graph was *y* = 0.0173*x* + 0.0195 (*R* = 0.9992). The extraction solution was used as a blank sample to calculate the limit of detection of Cr(VI). The value of the limit of detection (LOD) was calculated according to the following equation: LOD = 3SD_blank_/*b*, where *b* is the slope of the calibration graph. The limit of quantification (LOQ) was calculated as LOQ = 6SD_blank_/*b*. The LOD obtained for the extraction solution was 0.35 ng mL^−1^, while the LOQ was 0.71 ng mL^−1^. The volume of the extraction solutions and the mass of the soil samples were used for the calculations in order to evaluate these parameters for the soil samples. The LOD for Cr(VI) in soil was 17.5 μg kg^−1^, while the LOQ for soil was 35.5 μg kg^−1^.

The precision of measurements (expressed as the relative standard deviation (RSD)) was evaluated by analysing the standard solutions of Cr at a concentration of 4 ng mL^−1^ in extraction solution on the same day. The obtained value of RSD for measurements of six independent standards was 1.2%, which means that the precision of the measurements was satisfactory. The repeatability of extraction of chromium from the soil samples was evaluated by analysing extracts obtained by using the extraction solution at room temperature and heated to 90–95 °C. Repeatability was expressed as RSD for six independent extractions of the same sample. It was found that for both extraction procedures, the values of RSD were in the range of 1–10% for both analysed types of soil; however, slightly lower values were obtained for the mineral soil.

The trueness of extraction procedures of chromium was evaluated by analysing the certified reference material of soil CRM 041 (contaminated sandy clay soil) with a certified value of the Cr(VI) content determined by using the normalised EPA 3060A procedure (60 min of boiling the sample at 90 °C in a solution of 0.28 mol L^−1^ Na_2_CO_3_, 0.5 mol L^−1^ NaOH, 4 mol L^−1^ MgCl_2_ in 1 mol L^−1^ of phosphoric buffer). Good agreement of the content of Cr(VI) determined in 0.1 mol L^−1^ Na_2_CO_3_ extract obtained at room temperature (82.2 ± 5.5 μg g^−1^, *n* = 3) and after heating (86.6 ± 1.6 μg g^−1^, *n* = 3) with the certified value (86.3 ± 2.96 μg g^−1^) indicates good accuracy of the selected extraction procedures. The recovery of Cr(VI) in CRM 041 was 95.2 ± 6.3 and 100.4 ± 1.8%, respectively, which proved that the developed procedures may be applied for analysis of natural soil. We did not observed any influence of matrices of CRM 041 (as a mineral soil with 15 times lower content of manganese than the total content of chromium) on the recovery of Cr(VI) obtained after using extraction procedure at elevated temperature. It is worth noting that the developed procedures are simpler than normalised EPA 3060A in terms of the composition of the extraction medium (only Na_2_CO_3_ solution), conditions of extraction (room temperature) and time of extraction (10 min in the case of heating the suspension of the soil sample).

The uncertainty of measurements of Cr(VI) content in soil extracted with Na_2_CO_3_ solution by developed procedures were evaluated in accordance with the Guide to the Expression of Uncertainty in Measurement (2008), similarly to the scheme presented by Leśniewska et al. 2016. The modelling approach, based on a model equation, in which individual components of uncertainty that contribute to uncertainty of measurement are quantified, was used for estimation of combined standard uncertainty of measurements. The obtained expanded uncertainty (*U*) of measurements of Cr(VI) content in soil extracted with Na_2_CO_3_ solution at room temperature (82.2 ± 8.0 μg g^−1^; *U* = 9.7%, *k* = 2) was slightly higher than for extraction procedure at elevated temperature (86.6 ± 5.5 μg g^−1^; *U* = 6.3%, *k* = 2) due to a higher value of standard uncertainty of Cr(VI) recovery from CRM 041.

### Analysis of Soil Samples

Samples of soil (1–7) were collected from an industrially contaminated area of an old leather tannery where chromium(III) sulphate was used during the tanning process. The tannery had functioned there since the 1960s to the late 1990s. Samples 1, 2 and 3 were collected near the entrance to the wet, tanning and finishing departments (at a distance of 2–4 m from the building), respectively. Sample 4 was collected from the opposite side of the tannery building, probably along the route of transport of leather between the above-mentioned departments. Samples 5, 6 and 7 were collected at a distance of 1–2 m outside the tannery area. From all location, three mixed sub-samples were collected from a surface layer at a depth of 0–10 cm. All samples were categorised as mineral soil with various contents of organic matter and pH values (Table 3). The samples were classified mostly as non-contaminated soil, as the total content of chromium did not exceed its permissible limit for agricultural soil in Poland (Ordinance of the Minister of Environment of Poland 2016). Despite the use of large amounts of Cr(III) compounds in tannery, only two samples from its area (3 and 4) were classified as contaminated soil. The content of Cr(VI) in the samples was assessed by two developed and recommended procedures based on extraction with 0.1 mol L^−1^ Na_2_CO_3_ solution at room and elevated (90–95 °C) temperature. The results did not differ significantly (Table 3). The share of Cr(VI) in the total content of chromium in the analysed soil was very low, i.e. in the range of 0.8 to 4.5%. This low content of extracted Cr(VI) in the analysed soil, despite the high total content of chromium, indicates that chromium is present in very immobile forms, probably as stable Cr(III) compounds.

**Table 3:** The characteristic of soil samples collected from contaminated area of old leather tannery and the results of Cr(VI) extraction with Na_2_CO_3_ solution

Sample	pH_KCl_	Organic matter, %	Total content of Cr, μg g^−1^, *n* = 3	Extraction with Na_2_CO_3_ at room temperature, *n* = 3		Extraction with Na_2_CO_3_ at 90–95 °C, *n* = 3	
				Content of Cr(VI) ± SD, μg g^−1^	Percentage of Cr(VI) in total content ± SD, %	Content of Cr(VI) ± SD, μg g^−1^	Percentage of Cr(VI) in total content ± SD, %
Soil 1	7.8	1.86	68.6 ± 5.0	0.88 ± 0.03	1.29 ± 0.05	1.55 ± 0.06	2.27 ± 0.08
Soli 2	7.3	7.34	141.9 ± 6.1	3.54 ± 0.02	2.50 ± 0.02	4.36 ± 0.05	3.07 ± 0.03
Soil 3	7.2	10.14	283.9 ± 7.9	12.90 ± 1.43	4.54 ± 0.50	12.18 ± 0.83	4.29 ± 0.29
Soil 4	7.7	5.38	2336 ± 30	18.21 ± 0.50	0.78 ± 0.02	24.49 ± 0.73	1.05 ± 0.03
Soil 5	7.3	2.90	41.8 ± 3.2	0.54 ± 0.06	1.30 ± 0.14	0.45 ± 0.01	1.09 ± 0.03
Soil 6	7.4	3.10	26.0 ± 0.9	0.57 ± 0.05	2.20 ± 0.18	0.55 ± 0.02	2.13 ± 0.08
Soil 7	7.4	4.34	96.9 ± 2.8	2.38 ± 0.15	2.46 ± 0.16	1.94 ± 0.08	2.00 ± 0.08

## Conclusions

The procedures proposed in the literature for the extraction of various forms of Cr(VI) from solid matrices were tested under uniform conditions in order to evaluate their selectivity and efficiency towards Cr(VI) and Cr(III) species. None of the tested reagents showed suitable extraction power towards quantitative extraction of Cr(III) species. The procedures based on Na_2_CO_3_ (at room and elevated temperatures) were found to be suitable for extraction of Cr(VI) species from mineral soil. The procedure based on extraction of Cr(VI) with Na_2_CO_3_ solution at room temperature is recommended for organic soil analysis, as partial co-extraction and oxidation of Cr(III) arises at a higher temperature. The proposed procedures were validated and accuracy was proved by analysis of CRM of soil (CRM 041).

The studies conducted here show that the physical state of waste, initial form and oxidation state of chromium as well as soil properties had an influence on the final form of chromium species in soil, which affected its mobility. It was found that Cr(III) introduced into soil as a solid waste was immobile, while introduced as a liquid, it may became mobile depending on the environmental conditions. Cr(VI) compounds disposed in the form of solid waste enter the soil after solubilisation. Compounds of Cr(VI) disposed as liquid waste undergo various reactions with the soil matrix and their reduction may occur; therefore, data on the sources of pollution and original forms of chromium in wastes are necessary for better prediction and estimation of the presence of chromium species in soil.

The analysis of soil collected from the contaminated area of the old tannery revealed that the share of Cr(VI) was very low (only 0.8–4.5%) despite the high total content of chromium. This confirms that chromium was present in the soil in immobile forms, probably as Cr(III) compounds.
